# Wound Microenvironment Self-Adjusting Hydrogels with Thermo-Sensitivity for Promoting Diabetic Wound Healing

**DOI:** 10.3390/gels9120987

**Published:** 2023-12-17

**Authors:** Jia Li, Jing Guo, Bo-Xiang Wang, Yue Zhang, Qiang Yao, De-Hong Cheng, Yan-Hua Lu

**Affiliations:** 1 School of Textile and Material Engineering, Dalian Polytechnic University, Dalian 116034, China; lj18840597623@163.com (J.L.); nosferatu_1922@163.com (Q.Y.); 2 Liaoning Provincial Key Laboratory of Functional Textile Materials, Liaodong University, Dandong 118000, China; bxwang0411@163.com (B.-X.W.); zhangyue19875@163.com (Y.Z.); chengdehongldxy1@163.com (D.-H.C.); yanhualu@aliyun.com (Y.-H.L.); 3 School of Textiles and Garment, Liaodong University, Dandong 118003, China; 4 State Key Laboratory of Bio-Fibers and Eco-Textiles, Qingdao University, Qingdao 266071, China

**Keywords:** Antheraeapernyi silk gland protein, sodium alginate, poly(N-isopropylacrylamide), thermo-sensitive hydrogels, diabetic wound healing

## Abstract

The hard-healing chronic wounds of diabetics are still one of the most intractable problems in clinical skin injury repair. Wound microenvironments directly affect wound healing speed, but conventional dressings exhibit limited efficacy in regulating the wound microenvironment and facilitating healing. To address this serious issue, we designed a thermo-sensitive drug-controlled hydrogel with wound self-adjusting effects, consisting of a sodium alginate (SA), Antheraeapernyi silk gland protein (ASGP) and poly(N-isopropylacrylamide) (PNIPAM) for a self-adjusting microenvironment, resulting in an intelligent releasing drug which promotes skin regeneration. PNIPAM has a benign temperature-sensitive effect. The contraction, drugs and water molecules expulsion of hydrogel were generated upon surpassing lower critical solution temperatures, which made the hydrogel system have smart drug release properties. The addition of ASGP further improves the biocompatibility and endows the thermo-sensitive drug-controlled hydrogel with adhesion. Additionally, in vitro assays demonstrate that the thermo-sensitive drug-controlled hydrogels have good biocompatibility, including the ability to promote the adhesion and proliferation of human skin fibroblast cells. This work proposes an approach for smart drug-controlled hydrogels with a thermo response to promote wound healing by self-adjusting the wound microenvironment.

## 1. Introduction

The skin, being the largest organ in the body, is the first line of defense against injury and microbial invasion [1,2]. However, the structure and function of the skin can be affected by cuts, burns, surgical incisions or diabetes [3]. The chronic skin wound healing of diabetic patients becomes a challenging problem due to their damaged immune function and blood vessels [4,5]. Hydrogels have shown remarkable advantages in wound repair, are easy to make, and can be loaded with drugs, growth factors and other substances [6,7]. The traditional hydrogel diabetic wound dressing has good biocompatibility, degradability and good mechanical properties; it can absorb wound exudate to maintain the microenvironment of the wound area, and has drug release performance. However, the drug in traditional hydrogel wound dressing cannot achieve on-demand release [8,9].

Stimulus-responsive hydrogel diabetic wound dressing can achieve an on-demand release of drugs by absorbing wound exudate or changing its phase and volume in response to changes in the wound microenvironment, and can also achieve on-demand removal of diabetic wound dressings [10,11]. Thermo-sensitive bio-hydrogels in diabetic wound dressing are one of these smart hydrogels which can respond to changes in external ambient temperature without any chemical treatment of the internal structure [12,13]. Such bio-hydrogels can modulate and regulate feedback control of drug release. As a temperature-responsive polymer, Poly(N-isopropylacrylamide) (PNIPAM) has lower critical solution temperatures (LCST) that are extremely close to the human physiological temperature, about 32 °C [14,15]. When the ambient temperature is higher than the LCST, hydrogen bonds are formed between the isopropyl amide groups on the molecular chain of PNIPAM and the polymer contracts, releasing the drug and water for targeted release, which improves the drug utilization rate [16].

In terms of diabetic wound dressing material, sodium alginate (SA) is a naturally occurring polysaccharide biopolymer that is widely used in medicine, such as in slow release drugs [17,18], wound healing [19,20] and tissue repair [21,22]. It has strong hydrophilic properties, is low immunogenic and can absorb large amounts of liquid by exchanging calcium ions with sodium to form a salt-hydrophilic gel [23,24]. In addition, SA dressings have unique hemostatic properties, and can be used to treat deep wounds with severe exudation [25]. However, it is difficult to attach the dressing to the wound surface due to the weak adhesion of sodium alginate hydrogel. Moreover, SA hydrogel has poor mechanical properties, great brittleness and poor biodegradability, making it difficult to meet specific performance requirements of biological materials, limiting the range of applications for sodium alginate [26]. Antheraeapernyi silk gland fibroin (ASGP), derived from living, nontoxic organisms, has excellent biological affinity and cell adhesion properties not found in other biological proteins applied to wound dressing [27,28,29]. They have a unique, natural arginine-glycine-aspartate RGD tripeptide sequence that is capable of specific interactions with mammalian cells, and facilitates cell adhesion and growth [30,31,32,33]. ASGP can improve the adhesion, brittleness and biodegradability characteristics of sodium alginatehydrogel, which expands the application sphere of biomaterial wound dressing [34,35,36,37,38]. Based on this, the addition of PNIPAM endows the temperature-sensitive response of the gel dressing and results in a smart gel dressing with a temperature-sensitive drug release.

**Figure 1:**
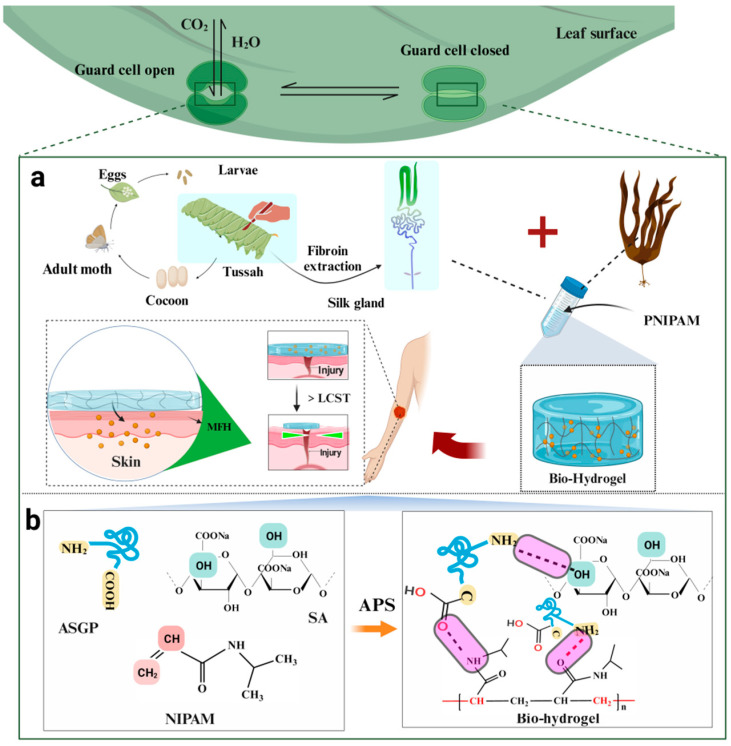
(**a**) Schematic diagram: the preparation of thermo-sensitive ASGP/SA/PNIPAM hydrogel containing MFH, and the stomata opening and closing of plant leaves affected by temperature; (**b**) substrate synthesis of thermo-sensitive hydrogel.

In the ecosystem, the channels through which water vapor is exchanged between the atmosphere and the leaves of plants are found in the stomata. The stomata is affected by temperature, with the stomata opening at the suitable temperature and closing at high temperatures [39]. The opening and closing of stomata due to temperature changes inspired our design of temperature-sensitive hydrogel wound dressings for smart controlled drug release (Figure 1).

Herein, we developed a series of thermo-sensitive ASGP/SA/PNIPAM drug-controlled hydrogels inspired by the variation of stomatal opening and closing with temperature (designated as thermo-sensitive ASGP/SA/PNIPAM hydrogel, Figure 1). The hydrogel was designed and synthesized using ASGP/SA as parent liquor, NIPAM as thermo-sensitive monomers and metformin hydrochloride as a drug model by in situ polymerization technology. The introduction of ASGP endows the hydrogel with good biocompatibility, adhesion and mechanical properties. Importantly, the PNIPAM has a good thermo-sensitive effect, regulating Metformin hydrochloride (MFH) drug release under different temperatures, which endows the hydrogel system with good thermo-sensitive controllable drug release properties. In vitro drug release evaluation was performed to confirm the smart controlled drug release effects of our proposed design principle, which is more conducive to improving the drug utilization and reducing their side effects. As prepared, the thermo-sensitive ASGP/SA/PNIPAM drug-controlled hydrogel is expected to be applied in the diabetic wound dressing field, and other tissue engineering fields.

## 2. Experimental Section

### 2.1. Materials

The fully grown 5th instar larvae of Antheraeapernyi were obtained from local farms (Dandong, China). Sodium alginate (SA, MW = 5.0 × 10^3^ kDa) was provided by Qingdao Bright moon Seaweed Group. (Qingdao, China). Metformin hydrochloride (MFH, ≥97% purity), N-isopropylacrylamide (NIPAM, ≥98% purity), N,N,N′,N′-Tetramethylethylenediamine (TEMED, ≥99% purity), N,N-MethylenebisacrylamideBis-acrylamide (Bis, ≥99% purity), Ammonium persulfate (APS, ≥98.5% purity), Sodium dodecyl sulfate (SDS, ≥99.5% purity), Tris (hydroxymethyl)aminomethane (Tris, ≥99.8% purity) and Human skin fibroblast (HSF) cells were purchased from Green Flag Biotechnology Development Co., Ltd. (Shanghai, China). Other reagents were all purchased from Shanghai Macklin Biochem Tech. (Shanghai, China). 

### 2.2. Preparation of Thermo-Sensitive ASGP/SA/PNIPAM Hydrogel

The preparation of thermo-sensitive ASGP/SA/PNIPAM hydrogel containing MFH was achieved by introducing NIPAM into a mixed solution of ASGP and SA (Figure 1). First, 4% ASGP solution of 5th instar Antheraeapernyi silk gland protein was prepared by dissecting silk glands from fifth instar larvae, then placing them in a mixed solvent of 1% SDS and Tris•HCl (0.01 M, pH = 8.0) solutions for 2 h, and centrifuging them to obtain the supernatant ASGP. Second, 15 mg MFH was added to the ASGP solution until sufficiently dissolved. Finally, the NIPAM (0.4 g/0.6 g/0.8 g/1.0 g/1.2 g), APS (0.8 g), 5% TEMED (160 uL), Bis (0.03 g) and 2mL 4% SA solution were separately added to 8 mL above ASGP solution, in turn. The aforementioned solution was mixed evenly and left to stand until the thermo-sensitive hydrogel was finished (A_8_S_2_/N_0.4_, A_8_S_2_/N_0.6_, A_8_S_2_/N_0.8_, A_8_S_2_/N_1.0_, A_8_S_2_/N_1.2_).

### 2.3. Morphological Characterizations

Scanning electron microscopy (SEM, JSM-IT100, Japan) was used to characterize the morphology. The structure and LCST of thermo-sensitive ASGP/SA/PNIPAM hydrogel was determined with Fourier transform infrared spectroscopy (FTIR, Spectrum Two, Waltham, MA, USA) and a differential scanning calorimeter (DSC, Q2000, New Castle, DE, USA), respectively. 

### 2.4. Mechanical Properties and Adhesion

The tensile/adhesion properties of different thermo-sensitive ASGP/SA/PNIPAM hydrogels were conducted with electric universal testing (AGS-X-10kN, SHIMADZU, Guangzhou, China). The aforementioned hydrogel was made into a uniform cylinder-shaped sample and tested. (1) Adhesion strength: Fresh pig skin was cut into 20 mm^2^ × 1 mm cylindrical splines, hydrogel was cut into 20 mm 2 × 1 mm splines, then the hydrogel was placed on the surface of the pig skin, another piece of pig skin was placed on the gel, and held at room temperature for 10 min. The bonding area was 20 mm × 20 mm, and the stretching rate was 5 mm/min. (2) Tensile performance: Cut the hydrogel into 20 mm× 50 mm× 0.03 mm splines and test at a rate of 2 mm/min until the spline breaks. (3) Compression performance: Cut the hydrogel into 20 mm^2^ × 50 mm cylindrical splines and test at a rate of 2 mm/min. Compress according to 50% compression mode.

### 2.5. Temperature Sensitivity

The macro morphologies of thermo-sensitive ASGP/SA/PNIPAM hydrogels at different temperatures were observed on camera. Before observation, the thermo-sensitive ASGP/SA/PNIPAM hydrogel was first stored at room temperature. The temperature changes of the hydrogel were observed using Infrared thermal imager (FLIR E8, Wilsonville, OR, USA), and the temperature data of hydrogel samples and heating plates were obtained. The hydrogel was cut into 20 mm^2^ × 1mm splines, then the hydrogel was placed on the surface of the heating plate.

### 2.6. Release of MFH

The dialysis method was used to test the MFH release from in vitro. Briefly, thermo-sensitive ASGP/SA/PNIPAM hydrogels (5.0 mg) were sealed in dialysis bags (MWCO 14,000), which were suspended in 100 mL of artificial intestinal fluid simulation solution (SIF, pH = 7.4) containing 0.05 M KH_2_PO_4_ and 0.0395M NaOH at different temperatures (20 °C, 37 °C). Afterward, 5.0 mL of SIF media was taken and replenished with the same volume of new medium at regular intervals. The samples were further analyzed, with the cumulative released amount of MFH in thermo-sensitive ASGP/SA/PNIPAM hydrogel at 230 nm.

The cumulative release amount (CR) of MFH in thermo-sensitive ASGP/SA/PNIPAM hydrogel was determined according to MFH standard curve:

(1) $$CR\left(\%\right)=\frac{C_{n}\cdot 100+5\sum_{i=0}^{n-1}C_{i}}{M_{0}}\times 100$$

where C*_n_*, C*_i_* is the concentration of MFH solution at the *n*th, *i*th time point; V_0_ = 0, C_0_ = 0. CR is one of cumulative release amounts of MFH averages. M_0_ is the mass of MFH into the thermo-sensitive ASGP/SA/PNIPAM hydrogel. 

### 2.7. Cytotoxicity Assay

The human skin fibroblast (HSF) cells were used as model cells to investigate the cytotoxicity of ASGP/SA/PNIPAM hydrogel. In a typical culture procedure, the logarithmic growth stage HSF cells were diluted and inoculated into 48-well plates for 3 days, then the old medium was replaced with 220 μL fresh culture medium, containing CCK-8 culture mixture (CCK-8:DMEM = 1:10). After further culturing for 1, 2, 5 and 7 days, 100 μL of the medium was sucked out to the 96-well plate, and the absorbance at 450 nm was recorded. The average value of the three reproes was taken as the final absorbance value of different groups and compared with the control group to calculate the cell survival rate:

(2) $$C.V.\left(\%\right)=\frac{{OD}_{s}}{{OD}_{c}}\times 100\%\;$$

where C.V. is cell survival rates, OD*s* is the OD value of sample extraction solution after cell culture and OD*c* is the OD value of the solution in the blank group, respectively.

## 3. Results and Discussion

### 3.1. Micro-Morphology and Characterization

**Figure 2:**
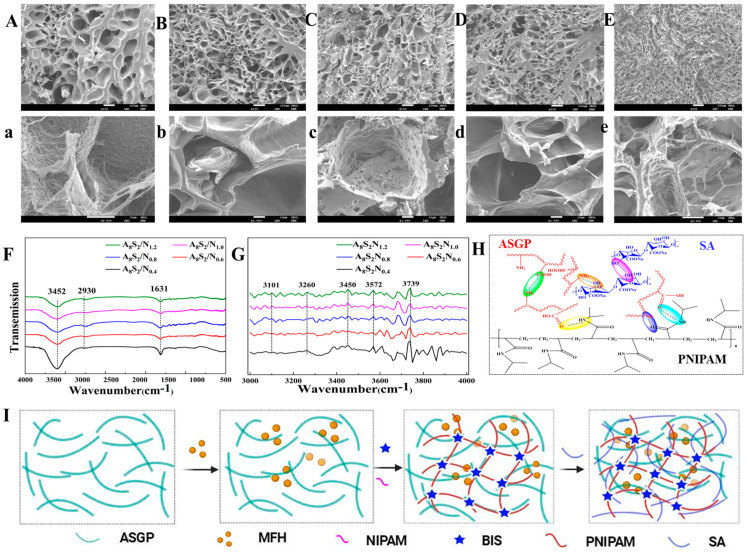
SEM images of ((**A**) ×100, (**a**) ×1000) A_8_S_2_N_0.4_, ((**B**) ×100, (**b**) ×1000) A_8_S_2_N_0.6_, ((**C**) ×100, (**c**) ×1000) A_8_S_2_N_0.8_, ((**D**) ×100, (**d**) ×1000) A_8_S_2_N_1.0_, ((**E**) ×100, (**e**) ×1000) A_8_S_2_N_1.2_, (**F**) FTIR spectra, (**G**) the second derivative spectra, (**H**) reaction mechanism and (**I**) substrate synthesis route of thermo-sensitive hydrogel.

Here, SEM was utilized to analyze the micro-morphology of the thermo-sensitive ASGP/SA/PNIPAM hydrogel. The hydrogel, as depicted in Figure 2A,a–E,e, possesses a porous structure with uniform parallel pores that facilitate oxygen delivery and offer the potential to expedite wound healing [40]. The increased content of NIPAM may cause the structure of hydrogel to be more uniform.

FT-IR spectroscopy (Figure 2F) is used to analyze the interaction between ASGP, SA and PNIPAM. The FTIR spectra of the thermo-sensitive ASGP/SA/PNIPAM hydrogel with different content of NIPAM are shown in Figure 2F. Figure 2F shows that there are obvious absorption peaks of thermo-sensitive ASGP/SA/PNIPAM hydrogel at 3452 cm^−1^, 2930 cm^−1^ and 1631 cm^−1^, which were related to −OH stretching vibration, −CH of six-membered ring stretching vibration and −COO− asymmetric stretching vibration, respectively [41]. The infrared spectra of thermo-sensitive hydrogel were smoothed using the Sa-vitzky–Golay method and second derivative spectra for studying the hydrogen bond interaction among SA, ASGP and PNIPAM, ranging from 3000 to 4000 cm^−1^ (Figure 2G). Figure 2G shows that there are obvious hydrogen bonds at 3101 cm^−1^, 3260 cm^−1^, 3450 cm^−1^, 3572 cm^−1^ and 3739 cm^−1^, which were related to hydrogen bond (Annular polymer), hydrogen bond (OH⋯O, etheric), intramolecular (OH⋯OH), intermolecular (OH⋯π) and free hydroxyl (−OH), along with ASGP, SA and PNIPAM, respectively [42,43]. According to the results of the FT-IR spectrum, the second derivative spectra and SEM, it is concluded that the abundant hydrogen bonds were formed between ASGP and SA, or ASGP and PNIPAM by the in situ polymerization method. The specific reaction mechanism and substrate synthesis route of thermo-sensitive hydrogel are shown in Figure 2H and Figure 2I, respectively.

### 3.2. Temperature Sensitivity

**Figure 3:**
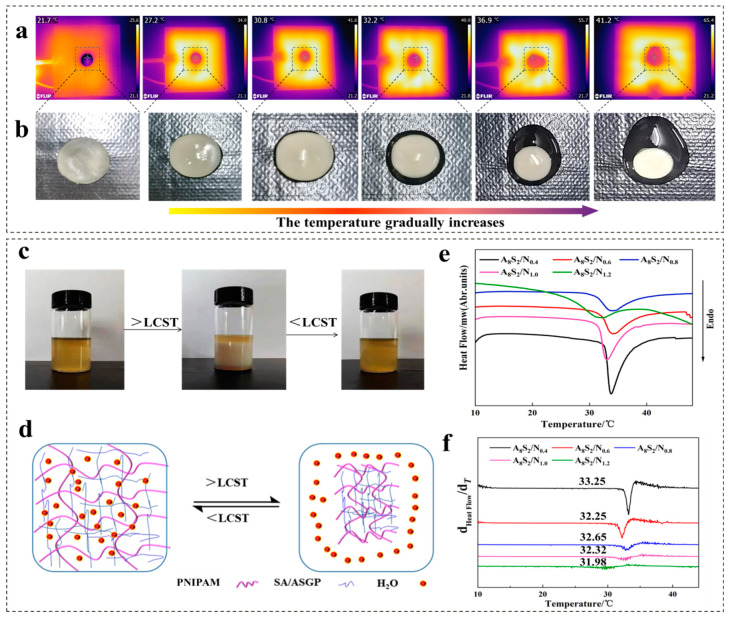
(**a**) The infrared image and (**b**) Macroscopic morphology of ASGP/SA/PNIPAM hydrogel as the external temperature varies from 20.7 °C to 41.2 °C; (**c**) Temperature-sensitive reversible and (**d**) Temperature-sensitive response mechanism; (**e**) DSC and (**f**) DSC second derivative of hydrogel.

The infrared camera was utilized to acquire the infrared image of ASGP/SA/PNIPAM hydrogel at varying temperatures (Figure 3). Figure 3a,b demonstrates the real-time changes in surface temperature and macroscopic morphology of ASGP/SA/PNIPAM hydrogel, as the external temperature varies from 20.7 °C to 41.2 °C. With the increase in temperature, the ASGP/SA/PNIPAM hydrogel undergoes a transition from opalescent transparency to white opacity, accompanied by a gradual reduction in overall sample size. Simultaneously, the ASGP/SA/PNIPAM hydrogel surface exhibited a gradual self-shrinkage phenomenon due to temperature-induced alterations in both hydrophobic interactions among ASGP/SA/PNIPAM hydrogel groups and hydrogen bonding between polymer chain segments [44].

The prepared thermo-sensitive hydrogel has reversible temperature sensitivity (Figure 3c). When the temperature falls below the LCST, the polymer chains within the ASGP/SA/PNIPAM hydrogel network become elongated and water molecules disperse throughout. When the temperature surpasses the LCST, there is an increase in intersegmental interactions within polymer chains, leading to a contraction of the ASGP/SA/PNIPAM hydrogel’s network structure. This results in the expulsion of water molecules, reduction in hydrogel size and subsequent occurrence of a gradual whitening phenomenon. Above all, the specific temperature-sensitive response mechanism of ASGP/SA/PNIPAM hydrogel is presented (Figure 3d).

Figure 3e,f shows the DSC and DSC second derivative of ASGP/SA/PNIPAM hydrogel. Based on the results of DSC and DSC second derivative analyses, the ASGP/SA/PNIPAM hydrogel exhibited a thermal transition temperature ranging from 31 °C to 34 °C. Furthermore, the thermal transition temperature of ASGP/SA/PNIPAM hydrogel decreased slightly with the increase of NIPAM content. This is because with the increase of thermo-sensitive monomer content, the space occupied by the thermo-sensitive polymer chain segment in the ASGP/SA/PNIPAM hydrogel network structure increases, and the sensitivity to temperature is enhanced [45,46]. The results above demonstrate the high temperature sensitivity of the prepared ASGP/SA/PNIPAM hydrogel.

### 3.3. Adhesion Ability

**Figure 4:**
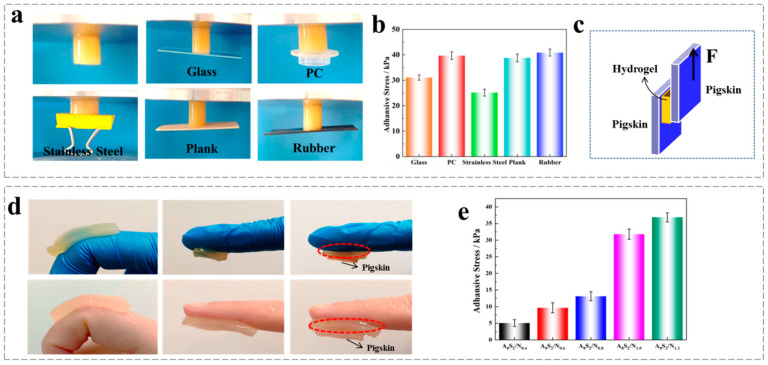
(**a**) Adhesion photo and (**b**) Adhesion strength of the thermo-sensitive ASGP/SA/PNIPAM hydrogel to glass, polycarbonate (PC), stainless steel, plank and rubber; (**c**) Adhesion photo of the hydrogel to pigskin; (**d**) Adhesion strength test diagram; (**e**) Adhesive strength of the hydrogel to pigskin.

In order to provide protection against external irritation, the wound dressing must be capable of adhering tightly to the skin tissue. Thermo-sensitive ASGP/SA/PNIPAM hydrogel, as a constituent of composite materials in direct contact with the skin, should exhibit excellent tissue adhesion. Therefore, the adhesion strength of thermo-sensitive ASGP/SA/PNIPAM hydrogel to pigskin was measured, and the test method is shown in Figure 4. The adhesion strength between hydrogel and various substrate surfaces was quantified using the aforementioned adhesion testing method.

Figure 4 shows that the thermo-sensitive ASGP/SA/PNIPAM hydrogel has good adhesion on a variety of substrates. The maximum adhesion strengths to glass, PC, stainless steel, plank and rubber were measured to be 31.48 ± 2.01 kPa, 39.87 ± 1.95 kPa, 25.056 ± 2.23 kPa, 38.56 ± 2.16 kPa, respectively (Figure 4a,b). Simultaneously, pig-skin viscosity experiments were performed on thermally sensitive ASGP/SA/PNIPAM hydrogel with different NIPAM contents, and the results showed that the hydrogel had good bonding properties. The hydrogel with different contents of NIPAM has a significant influence on the viscosity of thermo-sensitive hydrogel. With the increase of NIPAM, the viscosity of hydrogel is increased, and bond strength ranges from 5.07 ± 1.03 kPa to 36.87 ± 1.21 kPa, which is better than the 5 kPa of commercial wound dressing [47].

### 3.4. Mechanical Properties

**Figure 5:**
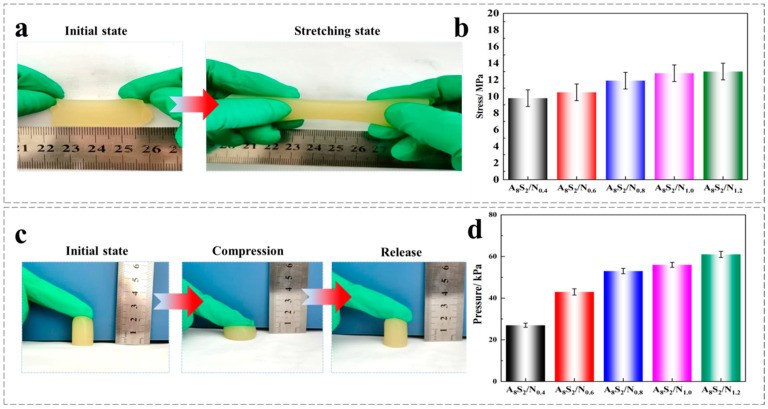
(**a**) Photo stretching of the thermo-sensitive ASGP/SA/PNIPAM; (**b**) Stretching of the hydrogel containing different contents of NIPAM; (**c**) Compression of the thermo-sensitive ASGP/SA/PNIPAM; (**d**) Compression of the hydrogel that was compressed to 50% of its initial height.

The mechanical properties of ASGP/SA/PNIPAM hydrogel were analyzed from a macroscopic perspective. The test results of hydrogel’s mechanical properties are shown in Figure 5. As can be seen in Figure 5a,b, the ASGP/SA/PNIPAM hydrogel can withstand 50% of extreme tensile without fracture; additionally, the ASGP/SA/PNIPAM hydrogel tensile strength slightly increases with the increase of thermo-sensitive monomer content. Furthermore, with the increase of thermo-sensitive monomer content, the compressive strength of ASGP/SA/PNIPAM hydrogel was significantly enhanced, and the maximum compressive strength was about 60 kPa (Figure 5c,d). This is because as the monomer content increases, the space occupied by the temperature-sensitive polymers in the ASGP/SA/PNIPAM hydrogel increases, which improves the interaction between the molecules and the hydrogen bonding so that the ASGP/SA/PNIPAM hydrogel has a uniform stress and load distribution during the deformation process [48].

### 3.5. Release of MFH 

**Figure 6:**
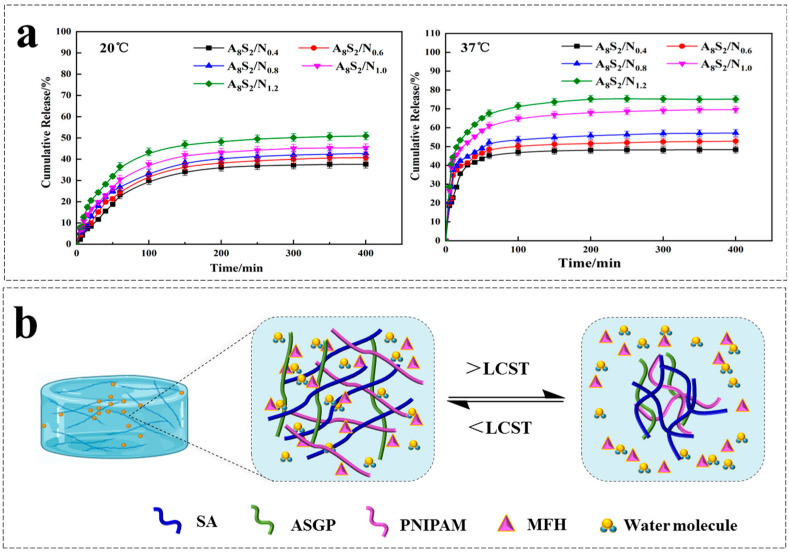
(**a**) Drug release of hydrogel; (**b**) The drug release performance of hydrogel.

Temperature is the “switch” that regulates the drug release rate of bionic intelligent hydrogel. As shown in Figure 6, the drug release behavior of temperature-sensitive hydrogel at different temperatures was studied. As can be seen in Figure 6a, the drug release rate of thermo-sensitive drug-carrying hydrogel gradually decreases with the extension of release time at 20 °C and 37 °C, and the cumulative drug release amount of hydrogel also increases with the increasing of heat-sensitive monomer content. This is because the higher the content of thermo-sensitive monomer, the stronger the hydrogen bond between the amide bond and water molecule in the thermo-sensitive monomer. As a result, the PNIPAM molecular chain is expanded, which is more conducive to drug release under the same drug release conditions [49,50].

The cumulative drug release amount of hydrogel can reach 75% at 37 °C, which is higher than that of its condition at 20 °C. This is due to the heightened hydrophobic effect of PNIPAM, resulting in its contraction and subsequent expulsion of drugs and water molecules upon surpassing LCST. Conversely, the hydrophobic effect of PNIPAM weakens, resulting in swelling of the hydrogel, with water molecules dispersing throughout when the temperature falls below LCST [51]. Specifically, the hydrogen bond between the amide group and water molecules in the hydrogel dominates, and the macromolecule of PNIPAM shows an extended state at low temperature. The hydrogen bond between the amide group and water molecules is weakened, and the interaction between the hydrophobic chain segments in the hydrogel is enhanced, resulting in the contraction of the PNIPAM molecular chain in the hydrogel network upon surpassing LCST [52,53]. Smart hydrogel has shown advantages in autonomously controlling drug release behavior based on ambient temperature.

In order to investigate the release mechanism of MFH, the diffusion kinetics of MFH in composite hydrogels was measured. The first order [54] empirical equation was fit for the cumulative drug release percentage:

*M_t_*/*M*_∞_ = 1 − e^−*kt*^ (3)

where *M_t_*/*M_∞_* is the percentage of drug released at time *t*th time point and *k* is the apparent drug release rate constant.

**Figure 7:**
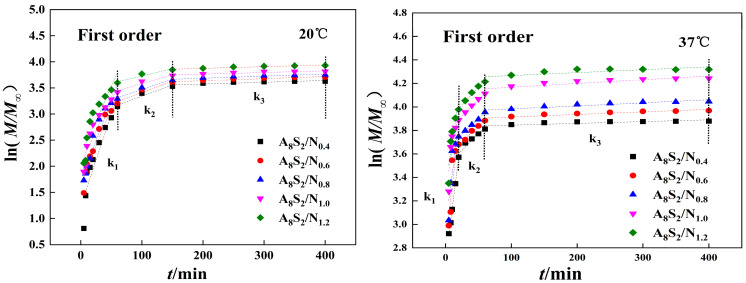
The fitting curve of hydrogels.

The fitting results of the dynamic model are shown in Figure 7 and Table 1. For 20 °C, the drug release of different ASGP/SA/PNIPAM hydrogels is divided into three stages (k_1_ > k_2_ > k_3_), and the drug release mainly occurs from 10 min to 150 min. For 37 °C, the drug release of different ASGP/SA/PNIPAM hydrogels is also divided into three stages (k_1_ > k_2_ > k_3_), and the drug release mainly occurs from 10 min to 60 min. Therefore, thermosensitive hydrogel wound dressings are susceptible to external temperatures and can release drugs at higher external temperatures to achieve the role of smart drug release.

### 3.6. Cytocompatibility In Vitro

**Figure 8:**
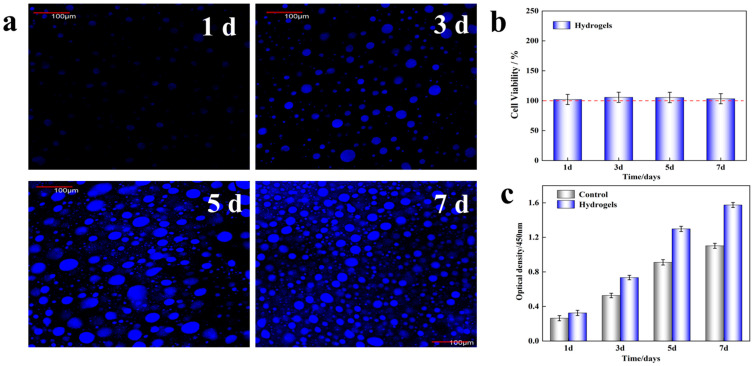
Cell assay analysis: (**a**) Live staining images of HSF cells after inoculating hydrogel after 1, 3, 5 and 7 days of culture; (**b**) Cell viability and (**c**) Proliferation of HSF cells measured by CCK-8 assays after 1, 3, 5 and 7 days of culture.

The low cytotoxicity of hydrogel wound dressing is a basic property demand for biomedical application [55,56,57]. To test the cytotoxicity of ASGP/SA/PNIPAM hydrogel, the live/dead staining of HSF cells with ASGP/SA/PNIPAM hydrogel was carried out during days one, three, five and seven. The Laser confocal scanning microscope was used to observe the morphology of HSF cells in ASGP/SA/PNIPAM hydrogel after one, three, five and seven days of culture, as shown in Figure 8a. As can be seen in Figure 8a, the HSF cells adhered and diffused well on the hydrogel sample on the third, fifth and seventh days. Each group showed favorable growth morphology, with the HSF cell number and HSF cell diffusion area increasing significantly with the increase of days. The HSF cells adhered to the surface of the ASGP/SA/PNIPAM hydrogel and showed good growth on the first day of culture. On the fourth and seventh days of culture, it can be seen that the HSF cells are uniformly distributed in the ASGP/SA/PNIPAM hydrogel, showing a 3D growth state; furthermore, the number of proliferating HSF cells increases significantly with increasing culture time. The results above indicate that HSF cells grow well in ASGP/SA/PNIPAM hydrogel, and have the effect of promoting cell proliferation.

At the same time, the CCK-8 test was carried to measure HSF cells viability after one, three, five, and seven days of culture (Figure 8b). As shown in Figure 8b, the results of the CCK-8 assay show that the viable cell rate maintains above 100%. The OD value of HSF cells increases gradually with the extension of culture time, and is higher than that of the blank group (Figure 8c). In conclusion, the ASGP/SA/PNIPAM hydrogel has inherent cytocompatibility. The ASGP/SA/PNIPAM hydrogel has non-negative effects on HSF cell viability and proliferation, and it has good biocompatibility.

## 4. Conclusions

In summary, we successfully fabricated a temperature-responsive ASGP/SA/PNIPAM drug-loaded hydrogel, which enables synergistic potential by modulating the wound microenvironment to play the role of a novel wound dressing. Compared to the previously reported research in hydrogel wound dressing, ASGP/SA/PNIPAM hydrogel has good temperature sensitivity and drug controllable release. In addition, the biological hydrogel showed the ability to promote the adhesion and proliferation of HSF cells. Therefore, this ASGP/SA/PNIPAM hydrogel with thermo-sensitivity and controllable drug release will provide a microenvironment that promotes wound regeneration and healing, and will have a high therapeutic effect on diabetic wound healing in future clinical practice. In future studies, we will conduct in vivo experimental research on diabetic wound models to build a more comprehensive theoretical and practical application basis. This will also provide theoretical support for further practical medical dressings.

## Figures and Tables

**Table 1:** Fitting parameters of three release dynamics models.

	First Order/20 s						First Order/37 °C					
	R_1_^2^	k_1_	R_2_^2^	k_2_	R_3_^2^	k_3_	R_1_^2^	k_1_	R_2_^2^	k_2_	R_3_^2^	k_3_
A_8_S_2_/N_0.4_	0.85	0.035	0.94	0.004	0.87	0.001	0.82	0.05	0.92	0.005	0.82	0.001
A_8_S_2_/N_0.6_	0.91	0.028	0.93	0.004	0.91	0.001	0.99	0.04	0.98	0.005	0.84	0.002
A_8_S_2_/N_0.8_	0.91	0.027	0.96	0.004	0.92	0.002	0.80	0.04	0.99	0.005	0.88	0.002
A_8_S_2_/N_1.0_	0.87	0.026	0.94	0.003	0.95	0.001	0.81	0.03	0.99	0.005	0.81	0.003
A_8_S_2_/N_1.2_	0.84	0.026	0.93	0.003	0.92	0.002	0.79	0.04	0.99	0.005	0.80	0.002

## Data Availability

Data will be made available on request.

## References

[1] Xie H., Bai Q., Kong F., Li Y., Zha X., Zhang L., Zhao Y., Gao S., Li P., Jiang Q. (2022). Allantoin-functionalized silk fibroin/sodium alginate transparent scaffold for cutaneous wound healing. Int. J. Biol. Macromol..

[2] Wu M., Gao B., Wei X. (2023). Recent advances in Raman spectroscopy for skin diagnosis. J. Innov. Opt. Health Sci..

[3] Rivero G., Meuter M., Pepe A., Guevara M.G., Boccaccini A.R., Abraham G.A. (2019). Nanofibrous membranes as smart wound dressings that release antibiotics when an injury is infected. Colloids Surf. A Physicochem. Eng. Asp..

[4] Lin H., Pan Y., Cai S. (2023). Direct Lineage Reprogramming for Induced Keratinocyte Stem Cells: A Potential Approach for Skin Repair. Stem Cells Transl. Med..

[5] Alderton G.K. (2014). Driving too much wound healing in the skin. Nat. Rev. Cancer.

[6] Rahmani F., Atabaki R., Behrouzi S., Mohamadpour F., Kamali H. (2023). The recent advancement in the PLGA-based thermo-sensitive hydrogel for smart drug delivery. Int. J. Pharm..

[7] Lee S., Santschi M., Ferguson S. (2021). A Biomimetic Macroporous Hybrid Scaffold with Sustained Drug Delivery for Enhanced Bone Regeneration. Biomacromolecules.

[8] Harmanci S., Dutta A., Cesur S., Sahin A., Gunduz O., Kalaskar D., Ustundag C. (2022). Production of 3D Printed Bi-Layer and Tri-Layer Sandwich Scaffolds with Polycaprolactone and Poly (vinyl alcohol)-Metformin towards Diabetic Wound Healing. Polymers.

[9] Yang L., Liang F., Zhang X., Jiang Y., Duan F., Li L., Ren F. (2022). Remodeling microenvironment based on MOFs-Hydrogel hybrid system for improving diabetic wound healing. Chem. Eng. J..

[10] Koffi D., Konan A., Ehouman E., Bonfoh B. (2023). Comparison of Chronic Wound Inpatients and Outpatients’ Diets and Meals Nutrient Content in Taabo Wound Management Unit, Côte d’Ivoire. Food Nutr. Sci..

[11] Falanga V., Isseroff R., Soulika A., Romanelli M., Margolis D., Kapp S., Granick M., Harding K. (2022). Chronic wounds. Nat. Rev. Dis. Primers.

[12] Wei Y., Feng X., Liu M., Huang X., Gao W., Wu H. (2022). Synthesis of low-molecular-weight gel with tunable gel-sol transition temperature for thermo-sensitive drug controlled release. J. Mol. Struct..

[13] Jeong B., Kim S., Bae Y. (2012). Thermosensitive sol-gel reversible hydrogels. Adv. Drug Deliv. Rev..

[14] Xu Y., Sun K., Zheng Q., Yao L., Dong Y., Song R. (2022). Synthesis and temperature-sensitive lubrication behavior of PNIPAM microgels for titanium alloy. Ind. Lubr. Tribol..

[15] Dhamecha D., Le D., Chakravarty T., Perera K., Dutta A., Menon J. (2021). Fabrication of PNIPAm-based thermoresponsive hydrogel microwell arrays for tumor spheroid formation. Mater. Sci. Eng..

[16] Lang X., Lenart W., Sun J., Hammouda B., Hore M. (2017). Interaction and Conformation of Aqueous Poly(*N*-isopropylacrylamide) (PNIPAM) Star Polymers below the LCST. Macromolecules.

[17] Yang Z., Fangv J., Tian D. (2022). 5-Fluorouracil-Loaded Sodium Alginate/Konjac Glucomannan Interacted with Attapulgite as a Potential Drug Delivery System. ChemistrySelect.

[18] Dalal S., Hussein M., Naggar E., Mostafa S., Dessuuki S. (2021). Characterization of alginate extracted from *Sargassum latifolium* and its use in *Chlorella vulgaris* growth promotion and riboflavin drug delivery. Sci. Rep..

[19] Li S., Wang X., Chen J., Guo J., Yuan M., Wan G., Yan C., Li W., Machens H., Rinkevich Y. (2022). Calcium ion cross-linked sodium alginate hydrogels containing deferoxamine and copper nanoparticles for diabetic wound healing. Int. J. Biol. Macromol..

[20] Soleimanpour M., Mirhaji S., Jafari S., Derakhshankhah H., Mamashli F., Nedaei H., Karimi M., Motasadizadeh H., Fatahi Y., Ghasemi A. (2022). Designing a new alginate-fibrinogen biomaterial composite hydrogel for wound healing. Sci. Rep..

[21] Yuan H., Zheng X., Liu W., Zhang H., Shao J., Yao J., Mao C., Hui J., Fan D. (2020). A novel bovine serum albumin and sodium alginate hydrogel scaffold doped with hydroxyapatite nanowires for cartilage defects repair. Colloids Surf. B Biointerfaces.

[22] Wen B., Dai Y., Han X., Huo F., Xie L., Yu M., Wang Y., An N., Li Z., Guo W. (2023). Biomineralization-inspired mineralized hydrogel promotes the repair and regeneration of dentin/bone hard tissue. npj Regen. Med..

[23] Keihan R., Radinekiyan F., Madanchi H., Aliabadi H., Maleki A. (2020). Graphene oxide/alginate/silk fibroin composite as a novel bionanostructure with improved blood compatibility, less toxicity and enhanced mechanical properties. Carbohydr. Polym..

[24] Ji D., Park J., Oh M., Nguyen T., Shin H., Kim J., Kim D., Park H., Kim J. (2022). Superstrong, superstiff, and conductive alginate hydrogels. Nat. Commun..

[25] Inata S., Sogawa H., Sanda F. (2023). Water-soluble alginate–based adhesive: Catechol modification and adhesion properties. Polym. J..

[26] Hu Z., An K., Wang J., Xu X., Chen Z., Hu J., Yang L. (2020). Preparation and Drug Release Behavior of Tussah Silk Fibroin Composite Membrane. Fiber Polym..

[27] Zhang J., Peng P., Chen L., Zhao L., Feng J. (2021). Antifouling poly(*N*-(2-hydroxyethyl)acrylamide)/sodium alginate double network hydrogels with eminent mechanical properties. Polym. Test..

[28] Wang Y., Wang X., Shi J., Zhu R., Zhang J., Zhang Z., Ma D., Hou Y., Lin F., Yang J. (2016). A Biomimetic Silk Fibroin/Sodium Alginate Composite Scaffold for Soft Tissue Engineering. Sci. Rep..

[29] Mandal B., Kundu S. (2010). Non-bioengineered silk gland fibroin protein: Characterization and evaluation of matrices for potential tissue engineering applications. Biotechnol. Bioeng..

[30] Mukherjee S., Krishnan A., Athira R., Kasoju N., Sah M. (2022). Silk fibroin and silk sericin in skin tissue engineering and wound healing: Retrospect and prospects. Natural Polymers in Wound Healing and Repair.

[31] Ma Y., Zeng W., Ba Y., Luo Q., Ou Y., Liu R., Ma J., Tang Y., Hu J., Wang H. (2022). A single-cell transcriptomic atlas characterizes the silk-producing organ in the silkworm. Nat. Commun..

[32] Hazra S., Nandi S., Naskar D., Guha R., Chowdhury S., Pradhan N., Kundu S., Konar A. (2016). Non-mulberry Silk Fibroin Biomaterial for Corneal Regeneration. Sci. Rep..

[33] Silva S., Gomes J., Vale A., Lu S., Rui L., Kundu S. (2020). Green pathway for processing non-mulberry antheraeapernyi silk fibroin/chitin-based sponges: Biophysical and biochemical characterization. Front. Mater..

[34] Rezaei F., Damoogh S., Reis R., Kundu S., Mottaghitalab F., Farokhi M. (2020). Dual drug delivery system based on pH-sensitive silk fibroin/alginate nanoparticles entrapped in PNIPAM hydrogel for treating severe infected burn wound. Biofabrication.

[35] Pacheco M., Kano G., Paulo L., Lopes P., Moraes M. (2020). Silk fibroin/chitosan/alginate multilayer membranes as a system for controlled drug release in wound healing. Int. J. Biol. Macromol..

[36] Keihan R., Khalili F., Aliabadi H., Maleki A., Bani M. (2020). Alginate hydrogel-polyvinyl alcohol/silk fibroin/magnesium hydroxide nanorods: A novel scaffold with biological and antibacterial activity and improved mechanical properties. Int. J. Biol. Macromol..

[37] Yin C., Han X., Lu Q., Qi X., Guo C., Wu X. (2022). Rhein incorporated silk fibroin hydrogels with antibacterial and anti-inflammatory efficacy to promote healing of bacteria-infected burn wounds. Int. J. Biol. Macromol..

[38] Pu Y., Wang P., Yang R., Tan X., Shi T., Ma J., Xue W., Chi B. (2022). Bio-fabricated nanocomposite hydrogel with ROS scavenging and local oxygenation accelerates diabetic wound healing. J. Mater. Chem. B.

[39] Lin C., Li Y., Sun X., Zhang X., Huang W., Ying J., Liu X., Hua M. (2020). Fabrication of superhydrophobic surfaces inspired by “stomata effect” of plant leaves via swelling-vesiculating-cracking method. Chem. Eng. J..

[40] Su Y., Zhang X., Wei Y. (2023). Nanocatalytic Hydrogel with Rapid Photodisinfection and Robust Adhesion for Fortified Cutaneous Regeneration. ACS Appl. Mater. Interfaces.

[41] Xu H., Shen L., Xu L., Yang Y. (2015). Low-temperature crosslinking of proteins using non-toxic citric acid in neutral aqueous medium:Mechanism and kinetic study. Ind. Crop. Prod..

[42] Li J., Wang B., Cheng D. (2022). Eco-Friendly Bio-Hydrogels Based on AntheraeaPernyi Silk Gland Protein for Cell and Drug Delivery. Gels.

[43] Gong X., Dang G., Guo J. (2019). Sodium alginate/feather keratin-g-allyloxy polyethylene glycol composite phase change fiber. Int. J. Biol. Macromol..

[44] Zu S., Wang Z., Zhang S. (2022). A bioinspired 4D printed hydrogel capsule for smart controlled drug release. Mater. Today Chem..

[45] Fei X., Lu T., Ma J., Zhu S., Zhang D. (2017). A bioinspired poly(*N*-isopropylacrylamide)/silver nanocomposite as a photonic crystal with both optical and thermal responses. Nanoscale.

[46] Varghese S., Lele A., Mashelkar R. (2000). Designing new thermoreversible gels by molecular tailoring of hydrophilic-hydrophobic interactions. J. Chem. Phys..

[47] Lih E., Lee J., Park K., Park K. (2012). Rapidly curable chitosan-PEG hydrogels as tissue adhesives for hemostasis and wound healing. Acta Biomater..

[48] Wang B., Zhang S., Wang Y. (2019). Regenerated *Antheraea pernyi* Silk Fibroin/Poly (*N*-isopropylacrylamide) Thermosensitive Composite Hydrogel with Improved Mechanical Strength. Polymers.

[49] Zhao J., Li S., Zhao Y., Peng Z. (2020). Effects of cellulose nanocrystal polymorphs and initial state of hydrogels on swelling and drug release behavior of alginate-based hydrogels. Polym. Bull..

[50] Patwa R., Zandraa O., Capáková Z., Saha N., Sáha P. (2020). Effect of iron-oxide nanoparticles impregnated bacterial cellulose on overall properties of alginate/casein hydrogels: Potential injectable biomaterial for wound healing applications. Polymers.

[51] Zu S., Wang Z., Zhang S., Guo Y., Chen C., Zhang Q., Wang Z., Liu T., Liu Q., Zhang Z. (2022). 4D printing of core–shell hydrogel capsules for smart controlled drug release. Bio-Des. Manuf..

[52] Mezhuev Y.O., Varankin A.V., Luss A.L., Dyatlov V.A., Tsatsakis A.M., Shtilman M.I., Korshak Y.V. (2020). Immobilization of dopamine on the copolymer of *N*-vinyl-2-pyrrolidone and allylglycidyl ether and synthesis of new hydrogels. Polym. Int..

[53] Pasban S., Raissi H. (2022). PNIPAM/Hexakis as a thermosensitive drug delivery system for biomedical and pharmaceutical applications. Sci. Rep..

[54] Bischofberger I., Trappe V. (2015). New aspects in the phase behaviour of poly-N-isopropyl acrylamide: Systematic temperature dependent shrinking of PNIPAM assemblies well beyond the LCST. Sci. Rep..

[55] Qian J., Ji L., Xu W., Hou G., Wang J., Wang Y., Wang T. (2022). Copper-Hydrazide Coordinated Multifunctional Hyaluronan Hydrogels for Infected Wound Healing. ACS Appl. Mater. Interfaces.

[56] Sun A., Hu D., He X., Ji X., Li T., Wei X., Qian Z. (2022). Mussel-inspired hydrogel with injectable self-healing and antibacterial properties promotes wound healing in burn wound infection. NPG Asia Mater..

[57] Sun A., He X., Li L., Li T., Qian Z. (2020). An injectable photopolymerized hydrogel with antimicrobial and biocompatible properties for infected skin regeneration. NPG Asia Mater..

